# A scoping review of magnetic resonance angiography and perfusion image synthesis

**DOI:** 10.3389/frdem.2024.1408782

**Published:** 2024-11-11

**Authors:** Rémi Lamontagne-Caron, Simon Duchesne

**Affiliations:** ^1^Centre de recherche de l'institut universitaire en cardiologie et pneumologie de Québec, Québec, QC, Canada; ^2^Département de médecine, Université Laval, Québec, Québec, QC, Canada; ^3^Département de radiologie et médecine nucléaire, Université Laval, Québec, QC, Canada

**Keywords:** review, perfusion, ASL, TOF-MRA, image synthesis, machine learning

## Abstract

**Introduction:**

Deregulation of the cerebrovascular system has been linked to neurodegeneration, part of a putative causal pathway into etiologies such as Alzheimer's disease (AD). In medical imaging, time-of-flight magnetic resonance angiography (TOF-MRA) and perfusion MRI are the most common modalities used to study this system. However, due to lack of resources, many large-scale studies of AD are not acquiring these images; this creates a conundrum, as the lack of evidence limits our knowledge of the interaction between the cerebrovascular system and AD. Deep learning approaches have been used in recent developments to generate synthetic medical images from existing contrasts. In this review, we study the use of artificial intelligence in the generation of synthetic TOF-MRA and perfusion-related images from existing neuroanatomical and neurovascular acquisitions for the study of the cerebrovascular system.

**Method:**

Following the PRISMA reporting guidelines we conducted a scoping review of 729 studies relating to image synthesis of TOF-MRA or perfusion imaging, from which 13 met our criteria.

**Results:**

Studies showed that T1-w, T2-w, and FLAIR can be used to synthesize perfusion map and TOF-MRA. Other studies demonstrated that synthetic images could have a greater signal-to-noise ratio compared to real images and that some models trained on healthy subjects could generalize their outputs to an unseen population, such as stroke patients.

**Discussion:**

These findings suggest that generating TOF-MRA and perfusion MRI images holds significant potential for enhancing neurovascular studies, particularly in cases where direct acquisition is not feasible. This approach could provide valuable insights for retrospective studies of several cerebrovascular related diseases such as stroke and AD. While promising, further research is needed to assess their sensitivity and specificity, and ensure their applicability across diverse populations. The use of models to generate TOF-MRA and perfusion MRI using commonly acquired data could be the key for the retrospective study of the cerebrovascular system and elucidate its role in the development of dementia.

## 1 Introduction

A complete and definitive understanding of the etiology of Alzheimer's disease (AD) remains elusive. Many hypotheses have been proposed, launching numerous studies of biomarkers at different stages of cognitive impairment in order to understand both its origin and future trajectory (Duchesne et al., 2023). While reports have long hypothesized that dysregulation of the cerebrovascular system is a likely initiator of neurodegeneration (Mann, 1985; Kalaria, 1992; de la Torre, 1999), recent empirical evidence has demonstrated how it is in fact a key co-morbidity in the early development of AD, preceding large scale amyloid deposits (Iturria-Medina et al., 2016). Therefore, the evaluation of cerebrovascular function appears crucial both for understanding and early detection of AD.

Magnetic resonance angiography (MRA) and perfusion imaging are the main techniques used for this purpose. MRA, particularly in the form of time-of-flight (TOF) imaging, provides detailed visualization of blood vessels, allowing the evaluation of vessel integrity and extracting vessel morphology, such as the diameter, volume and surface without the need for contrast agents (Laub, 1995; Miyazaki and Akahane, 2012; Wheaton and Miyazaki, 2012). Perfusion imaging for its part measures dynamic aspects of cerebral blood flow (CBF), cerebral blood volume (CBV), and mean transit time (MTT), providing insight into the brain's hemodynamic state. Multiple MR techniques map perfusion, such as arterial spin labeling (ASL), a non-invasive acquisition allowing for the calculation of the CBF, dynamic susceptibility contrast MR (DSC-MRI) and dynamic contrast-enhanced MR, both based on the use of an injected contrast agent (Parkes et al., 2004; Essig et al., 2013; Gaillard, 2016; Koenig et al., 1998; Petrella and Provenzale, 2000). Computed tomography (CT) perfusion and positron emission tomography imaging can also be used for this purpose (Ueda et al., 1994; Koenig et al., 1998; Ueda et al., 1999; Wachtel et al., 2001). These measurements are crucial for identifying abnormalities in blood flow that may contribute to the pathogenesis of neurodegenerative diseases such as Alzheimer's.

However, TOF-MRA and perfusion imaging both require significant resources, including access to high-field MRI scanners (typically 1.5 or 3T), which are necessary for acquiring high-resolution images and trained personnel that are essential for acquisition and analysis. Furthermore, the time needed for each scan can be upwards of tens of minutes depending on the acquisition protocol, which is even more significant in large imaging studies where optimizing participants time is essential and the use of contrast agent is not recommended when possible (Wheaton and Miyazaki, 2012; Miyazaki and Akahane, 2012; Albert et al., 2010; Parkes et al., 2004). This, without mentioning the recency of some of these techniques, such as ASL, which makes their availability scarce and the difficulties in ensuring standardization of imaging across scanners in a multi-centric setting. For these reasons, many large studies, such as ADNI 1, ADNI 2, ADNIGO, the COMPASS-ND and the UK Biobank, have historically not included some of these modalities, even though MRI scanners would allow such acquisition (Jack et al., 2008; Albert et al., 2010; Smith et al., 2014; Gunter et al., 2017; Chertkow et al., 2019). On the other hand, modalities like T1-weighted (T1-w), T2-weighted (T2-w), fluid-attenuated inversion recovery (FLAIR), T2* or susceptibility weighted imaging (SWI) and others, are present in most imaging studies relating to AD (e.g., LaMontagne et al., 2019; Smith et al., 2014; Jack et al., 2008; Gunter et al., 2017; Albert et al., 2010). These are necessary if one wishes to measure cortical atrophy, detect white matter lesions and other cerebrovascular abnormalities (Abrigo et al., 2023; Luo et al., 2019; Prosser, 2024). Thus, given the emphasis on other hypotheses, there exists a conundrum in that evidence to either support or infirm the role of cerebrovascular perfusion in dementia remains scarce, which drives the exclusion of such sequences in acquisition protocols due to limited scanning time.

The capacity to generate synthetic estimates of these image types from other, more easily obtained contrasts (e.g., T1-weighted, T2-weighted or FLAIR) would unlock the study of cerebrovascular deregulation in a number of legacy, retrospective studies; while possibly allowing prospective studies to save valuable scanning time by not including these contrasts in their imaging protocols.

Although image generation is a new field of research, the application of these techniques to medical image synthesis has recently started seeing significant leaps with the introduction of generative adversarial networks (GANs) in 2014 and U-nets in 2015 (Goodfellow et al., 2014; Ronneberger et al., 2015; Kazeminia et al., 2020; Yi et al., 2019) (see extensive review by Ali et al., 2022). Similarly, Transformers have shown great potential in generating accurate and precise images, outperforming convolutional networks in tasks such as tumor segmentation (Vaswani et al., 2017; Abu-Srhan et al., 2021; Zhao et al., 2022; Manzari et al., 2023). One of their key advantages lies in attention mechanisms, which mirror aspects of human vision (Manzari et al., 2023; He et al., 2022; Shamshad et al., 2023). These mechanisms allow the model to focus on specific areas of an image by assigning variable importance to different regions, effectively filtering out noise (Bahdanau et al., 2016; Vaswani et al., 2017; He et al., 2022). This not only improves accuracy but also makes the models more interpretable than standard convolutional models, which is a significant asset in health science (Bahdanau et al., 2016; Vaswani et al., 2017; He et al., 2022). Typical Transformer structure enables it to efficiently handle large amounts of data while overcoming the limitations of convolutional models, such as their difficulty in capturing non-local information and distant pixel correlations (Vaswani et al., 2017).

To our knowledge, no review has yet been published to date on the generation of images of the cerebrovasculature and its function. Therefore, our objective was to review the state of the art in this field, with specific attention to the comparison of the types of MRI input data, the different learning architectures, and the choice of metrics used for assessing the accuracy of synthesis.

## 2 Method

### 2.1 Eligibility criteria

To be included in this review, the studies needed to be original research papers, published in English, reporting on either “perfusion imaging” or “TOF-MRA”, and include terms relating to “medical image synthesis” and “machine learning”. Papers were not excluded based on date of publication, but image synthesis being a fairly novel field of research (Goodfellow et al., 2014), studies were expected to have been published after 2014. All forms of inputs used to generate images were accepted; likewise for all types of participants (e.g., with or without cognitive decline). Exclusion criteria were studies generating the wrong image (i.e., non TOF-MRA and non perfusion images), using the wrong organ (non-brain or non-human images), and with the wrong outcome, such as not generalizable outside their training data.

### 2.2 Information sources and search strategy

This scoping review followed the PRISMA reporting guidelines (Page et al., 2021). The study was conducted in the PubMed database in October 2023. Since machine learning is a quickly evolving field, a similar search was conducted in two pre-publication repositories, arXiv and medRxiv. The keywords used for the search were “((perfusion imaging) OR (arterial spin labeling)) AND (image synthesis) AND ((machine learning) OR (deep learning) OR (Artificial intelligence) OR (neural networks))” and “(TOF-MRA) AND ((synthetic) OR (machine learning) OR (synthesis))”.

### 2.3 Study selection process

Abstracts and articles management for the review was performed with the software Covidence (Innovation, 2023) by both authors as reviewers. Once the initial search was completed, abstracts were uploaded to Covidence and duplicates removed. Both reviewers performed a screening process based on abstracts, followed by a full-text review during which articles were included or excluded according to the criteria mentioned above. Conflicts were resolved at a consensus conference between reviewers.

### 2.4 Data extraction

Data was extracted by one of the reviewers (RLC) using a Covidence data extraction spreadsheet made for this review. The basic characteristics of the study included the authors, date of publication, the studied population (sex, age, number of subjects, cognitive status) and data acquired or used during the study. These data are reported in Table 1. Furthermore, information about the methodology used to generate the images was obtained: the type of machine learning architecture, training metric(s), validation method(s), type(s) of input to the network, as well as network output(s).

**Table 1:** Study characteristics.

**ID**	**References**	**Output**	**Dataset**
**Perfusion studies**			
1	Hess et al., 2019	CBF, CBV, TTP, MTT, Tmax	–
2	Huang et al., 2019	ASL	–
3	Zhang et al., 2020	CBF, CBV, tBAT, T1, B1	–
4	Li et al., 2021	ASL	ADNI-1
5	Asaduddin et al., 2023	CBF, CBV, MTT, Tmax	–
6	Gava et al., 2023	CBF, CBV, TTP	–
7	Kossen et al., 2023	CBF, CBV, MTT, Tmax, TTP	Heidelberg (*n* = 204) PEGASUS (*n* = 80)(*n* = 72)
**TOF-MRA Studies**			
8	Olut et al., 2018	TOF-MRA	IXI (*n* = 440)
9	Fujita et al., 2020	TOF-MRA	-
10	Kossen et al., 2021	TOF-MRA Segmentation label	PEGASUS (*n* = 66) 1000plus (*n* = 55)
11	Subramaniam et al., 2022	TOF-MRA Segmentation label	PEGASUS (*n* = 72) 1000plus (*n* = 65)
12	You et al., 2022	TOF-MRA	-
13	Kossen et al., 2022	TOF-MRA Segmentation label	PEGASUS (*n* = 66) 1000plus (*n* = 65)

This table shows what modality is generated by each model in the section *Output*. Studies generated either CBF, cerebral blood flow; CBV, cerebral blood volume; MTT, mean transit time; TMax, time-to-maximum; TTP, time-to-peak; ASL, arterial spin labeling; TOF-MRA or segmentation labels of arteries. the *Dataset* column shows several publicly available databases were used, such as ADNI-1, Heidelberg, IXI, PEGASUS and 1000plus, while studies marked by “–” used in-house data or did not disclose the origin of the data.

### 2.5 Synthesis method and quality assessment

Results of the scoping review are presented in Tables with the description of the academic work. However, for this review, no strict bias assessment scale was used, since most studies only reached the algorithm stage, data and algorithm biases were assessed using the framework of bias in machine learning (Mehrabi et al., 2021), specifically the “data to algorithm” phase of development. The quality of each study was assessed on sample size used for training, the use of standard validation tasks and representation of the data.

## 3 Result

### 3.1 Study selection

**Figure 1:**
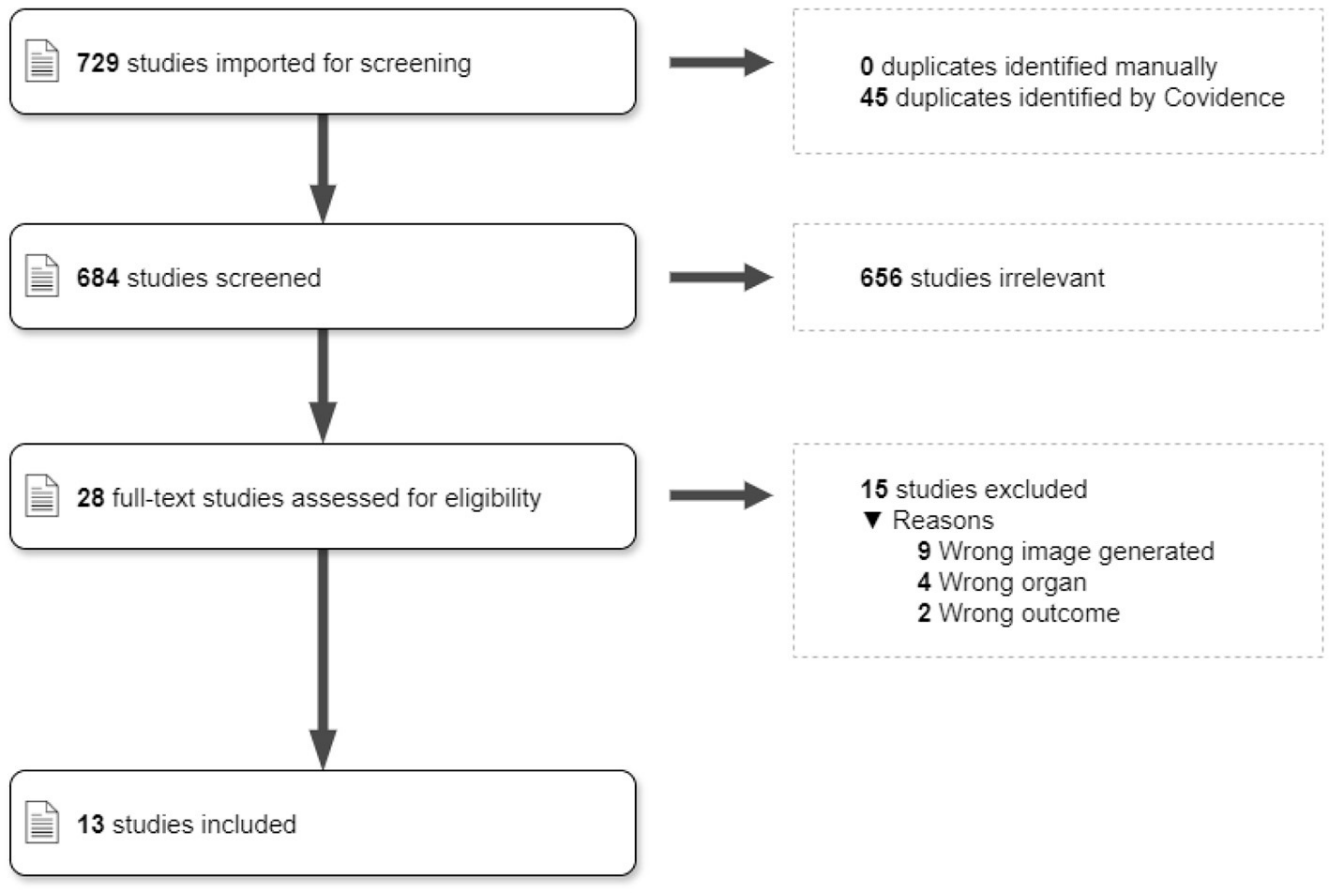
PRISMA diagram. The description of study selection through Covidence, following the PRISMA protocol.

Seven hundred and twenty-nine studies were uploaded to Covidence from the PubMed, medRXiv, and arXiv databases. From these, 45 duplicates were removed. During the review of the title and abstract, 656 articles were deemed irrelevant, leaving 28 papers for the full-text review, which excluded a further 15 articles. For the most part, papers were excluded for having the wrong study design (e.g., doing segmentation instead of image generation; or generating images on other organs than the brain), leaving 13 papers for data extraction and reporting (seven on perfusion and six on MRA synthesis). The selection process is represented in Figure 1.

### 3.2 Study characteristics

Study characteristics are presented in Table 1. Overall, we found thirteen studies relating to the generation of perfusion maps, ASL or TOF-MRA images. Seven studies were related to the generation of perfusion maps, either synthesizing cerebral blood flow (CBF), cerebral blood volume (CBV), mean transit time (MTT), time-to-maximum (TMax), time-to-peak (TTP), or a combination of these maps. For the studies generating TOF-MRA, Kossen et al. (2021), Subramaniam et al. (2022), Kossen et al. (2022) also synthesized arteries' segmentation labels while the other two only generated images. Finally, one paper generated ASL images.

The studies were conducted by nine different research teams. The first team (Kossen and colleagues) published four papers; the Huang research team published Li et al. (2021) and Huang et al. (2019) and all other teams had *n* = 1 paper. Regarding data provenance, most studies used in-house datasets or did not specify the origin of the data (*n* = 5). Of the studies that used databases, four used the PEGASUS database, containing subjects affected by steno-occlusive disease (Mutke et al., 2014), three used the *1000plus* database, with patients admitted to ER with acute stroke (Hotter et al., 2009), and one used a dataset acquired at the Heidelberg University Hospital, also including patients with acute stroke (Kossen et al., 2023).

### 3.3 Subjects characteristics

While three of the papers studied neurologically healthy subjects, three studies worked on patients with unspecified cerebrovascular disease, and other studies focused on patients with dementia (*n* = 2), acute ischemic stroke (*n* = 4), steno-occlusive disease (*n* = 1), Moyamoya disease (*n* = 1), or intracranial aneurysms (*n* = 2), as shown in Table 2.

**Table 2:** Subjects characteristics.

**ID**	**References**	**Population description**	**Age [years]**	**Sample size**	**F/M**
1	Hess et al., 2019	Acute ischemic stroke	–	151	–
2	Huang et al., 2019	MCI, AD and healthy	70.56 ± 7.20	355	–
3	Zhang et al., 2020	Neurollogically healthy Moyamoya disease	26 ± 4 36.3 ± 0.9	10	6/4
4	Li et al., 2021	MCI, AD and healthy	70.56 ± 7.20	355	–
5	Asaduddin et al., 2023	Ischemic stroke with occlusion or stenosis	30-73	60	31/29
6	Gava et al., 2023	Acute ischemic stroke	–	115	–
7	Kossen et al., 2023	Stroke Steno-occlusive disease	–	276	–
8	Olut et al., 2018	Neurologically healthy	–	440	–
9	Fujita et al., 2020	Neurologically healthy Intracranial aneurysms	27.4 ± 4.2 69.7 ± 6.1	15	3/12
10	Kossen et al., 2021	Cerebrovascular disease	–	121	–
11	Subramaniam et al., 2022	Cerebrovascular disease	–	137	–
12	You et al., 2022	Intracranial aneurysms	60 ± 11	377	293/84
13	Kossen et al., 2022	Cerebrovascular disease	–	131	–

This table shows the diagnostic status of the studied population in the column *population description*, the age distribution in column *age*, the total number of subjects in *Sample size* and the female to male ratio in the column *F/M*. For the age, data is provided in the form *mean*±*std* for all but one study where the statistic was only provided as a range.

Most studies did not report participants' age and sex. In fact, only Zhang et al. (2020), Asaduddin et al. (2023), Fujita et al. (2020), You et al. (2022) mentioned the male to female ratio in their datasets. The same is true for participant's age, with only Huang et al. (2019), Zhang et al. (2020), Li et al. (2021), Asaduddin et al. (2023), Fujita et al. (2020) reporting age averages or range. Finally, only Hess et al. (2019) and Gava et al. (2023) explicitly mentioned exclusion criteria for their participants. For example, Hess et al. (2019) excluded 38 cases where their arterial input function was inaccurate while Gava et al. (2023) excluded 12 time series with no or low contrast, images with excessive artifacts due to participant's movement, or premature termination of the acquisition. It should be noted that most studies using existing datasets tend to refer to the original study for any information about the data, rather than reporting salient information themselves, including quality control results.

### 3.4 Network characteristic

Deep learning was used by all authors to generate images in a variety of architectures. The most common method used was GAN, with eight articles using a variation of this model, followed by U-nets (*n* = 2), convolutional neural networks (CNN; *n* = 2), residual networks (ResNet; *n* = 1) and simple neural network (NN; *n* = 1). It is of note that some definitions overlap between architectures. For example, at a high level, a ResNet, a U-net, and a GAN are all CNNs or use convolutions in some way. In this paper, following the authors' convention, we will use the most differentiating definition, meaning GANs using U-net will be referred to as GANs, ResNet using CNN referred to as ResNet, and so forth.

Multiple variations of GAN models were used. We will first review the three studies by Kossen and colleagues that explored various GAN configurations. In Kossen et al. (2023) they first used a model with a time convolution, to generate perfusion maps with lower error rate and higher peak signal to noise ratio PSNR than images produced with a standard pix-2-pix (p2p) GAN. PSNR and SNR are metrics for evaluating the quality of synthetic images by measuring the clarity of the synthetic image compared with the level of the signal in the real image. In image synthesis, higher SNR values indicate that the model successfully minimizes noise in the generated images, improving their overall quality. In Kossen et al. (2021) the authors compared a traditional GAN with a Wasserstein-GAN (WGAN) and its variations with gradient penalty and spectral normalization. In short, the WGAN is a novel network architecture which uses different optimization methods, allowing the training to be more stable and reducing the vanishing gradient problem (Arjovsky et al., 2017). They concluded that the WGAN with gradient penalty and spectral normalization yielded images with the best quality (FID of 37.01) and most accurate segmentation labels (Dice score of 0.85), compared to traditional GAN (FID: 141.82; Dice score: 0.79) (Kossen et al., 2021). The follow-up study, Kossen et al. (2022), obtained worst results (average FID of 62) while investigating the use of GAN for data anonymization. This was likely due to the anonymization factor impacting image quality and realism (Kossen et al., 2023). Outside of this research team, the paper by Subramaniam et al. (2022) demonstrated more accurate results by using 3D vs. 2D GAN. This paper also uses the FID score as a validation metric, but the value was computed from the MedicalNet model (Chen et al., 2019), a ResNet trained on medical images instead of on the commonly used Inception-v3 model (Szegedy et al., 2015). Using this, they recorded a FID of 0.0206 and a Dice score of 0.841 for their best model with spectral normalization and mixed precision model. Li et al. (2021) used a variational auto-encoder (VAE)-GAN to generate perfusion maps from T1-weighted (T1-w) images. Using these synthetic images they managed to improve the diagnosis of AD in patients without perfusion images by 43% in machine learning models. Finally, the steerable filter GAN model from Olut et al. (2018) demonstrated the possibility of synthesizing TOF-MRA from T1- and T2-w images. The filter is designed to help the model emphasize vascular structure, in doing so the obtained higher PSNR and Dice score than comparable models without steerable filter.

The study from You et al. (2022) used the cycleGAN, an unsupervised machine learning method that allows image-to-image translation (Zhu et al., 2020), to generate TOF-MRA with very low background noise. In fact, overall image quality, sharpness and vessel appearance were on average higher in the synthesized TOF-MRA than the original training PETRA images. A visual analysis by 17 radiologists also did not show significant differences in diagnostic power between synthetic and normal TOF-MRA. The peak signal to noise ratio (PSNR) was also high with 17.51 dB on average and a structural similarity index measurement (SSIM) of 0.71 ± 0.02. For perfusion maps, Asaduddin et al. (2023) generated images with SSIM of 0.87 ± 0.08 an PSNR of 27 ± 4 dB using GAN. On the other hand, their U-net generated more accurate images [higher PSNR and lower root mean square error (RMSE)], but with lower structural similarity (Asaduddin et al., 2023). Similarly, Kossen et al. (2023) obtained SSIM as high as 0.986 and PSNR as high as 42 dB, using a GAN with a time convolution to generate perfusion maps.

The second most used neural network was the U-net. Fujita et al. (2020) used such a network to generate TOF-MRA from 3D quantitative synthetic MRI. In combination with a single convolution layer, they were able to generate TOF-MRA for healthy subjects and aneurysm patients, despite the training data containing only healthy subjects. Furthermore, the synthetic images had a better signal-to-noise ratio than the real TOF-MRA, with PSNR as high as 35.3, and the extraction of arterial vessels showed no difference between the real and synthetic TOF-MRA, except for small vessels (Fujita et al., 2020). The SSIM of 0.93 and a high frequency error norm of 0.86 also shows high accuracy when compared to the ground truths. Using radiologists to manually segment the infarct core and penumbra, Gava et al. (2023) showed no significant difference between synthetic perfusion maps and real perfusion maps. Moreover, they showed an acceptable (>0.7) match between all ground truth images and their corresponding synthetic images and the lesions' volume were also highly correlated (>0.98) between synthetic and real images (Gava et al., 2023).

Finally, the CNN from Hess et al. (2019) closely resembling a U-net (down-sampling followed by up-sampling with cross connections), but they first applied a convolution on the time axis of the sequences. Using this model, they obtained mixed results, with some synthetic maps close to the ground truth and others very far due to various reasons, such as unregistered images. Overall, the best model had a mean absolute error of 0.513 (for values bounded between 0 and 20). These results were significantly worse than the GAN model from *Kossen* with a MAE of 0.015 for the same task, the generation of Tmax maps (Hess et al., 2019). The model from Zhang et al. (2020) is a fully connected voxel-wise neural network to generate different perfusion maps from fingerprinting-ASL, a sequence of 500 images using different parameters (labeling duration and label-control acquisition order). Without using CNN they managed to generate images with comparable parameter values to the ground truth in both gray and white matter. Synthetic values were also shown to be related with their corresponding images with a coefficient of determination ≥0.65 for CBF, CBV, *T*_1_ and bolus arrival time. Synthetic images were also used to identify high bolus arrival time and low CBF regions consistent with obstructed arteries in Moyamoya disease patients, even though this type of data was not used during training (Zhang et al., 2020).

### 3.5 Input image description

From Table 3, we see that the studies reviewed utilized nine different types of input data: noise vector (*n* = 3), DSC-MRI (*n* = 2), T1-w (*n* = 2), T2-w (*n* = 1), MR fingerprinting arterial spin labeling (MRF-ASL) (*n* = 1), contrast-enhanced time-resolved dynamic MR angiography (DA-MRA) (*n* = 1), CT perfusion (*n* = 1), 3D quantification using an interleaved look locker acquisition sequence (3D QALAS) MRI (*n* = 1) and pointwise encoding time reduction with radial acquisition MRA (PETRA-MRA) (*n* = 1). The most common type of data used was noise vectors, employed in models by Kossen and colleagues for vessel segmentation and image synthesis. These were used since the purpose of the models were to generate images for data augmentation and thus did not need to translate from another image. Additionally, DSC-MRI, used in studies such as Hess et al. (2019) and Kossen et al. (2023), provides perfusion data, capturing CBF dynamics, cerebral blood volume (CBV), time to peak (TTP), mean-transit time (MTT) and max arrival time (Tmax) using contrast agent. Similarly, other raw perfusion images were used e.g., MRF-ASL in Zhang et al. (2020), and CT perfusion in Gava et al. (2023). These models were used to bypass the traditional methods of processing these images into perfusion maps, as in the case on Hess et al. (2019) where they tried to make an end-to-end model with some success. Two studies used different MRA modalities to generate TOF-MRA or perfusion maps. Indeed, Asaduddin et al. (2023) used DA-MRA, an agent-based contrast used to visualize arteries, by subtracting a baseline image to a time-series of contrast agent enhanced images and You et al. (2022), where they use the PETRA-MRA sequence, a contrast utilizing ultra-short echo times which can display blood vessels near or in hard-to-image areas such as the skull base.

**Table 3:** Network characteristic.

**Author**	**Architecture**	**Metrics**	**Input image**	**Inputresolution[*mm*^3^]**	**Outputresolution[*mm*^3^]**	**Scannerstrength**
Hess et al. (2019)	CNN TC	MAEC	DSC-MRI	–	–	–
Huang et al. (2019)	ResNet	PVE correction CBF computing CBF ANOVA	T1-w	3 × 3 × 5	3 × 3 × 5	3 T
Zhang et al. (2020)	Regression NN	SNR Corr *R*^2^	MRF-ASL	–	–	3 T
Li et al. (2021)	VAE-GAN	AD diagnostic	T1-w	–	–	3 T
Asaduddin et al. (2023)	U-net p2p GAN	RMSE PSNR SSIM Segmentation	DA-MRA	1.6 × 1.2 × 0.9	1.8 × 1.8 × 4	3 T
Gava et al. (2023)	U-net	Segmentation	CT perfusion	–	–	80 kV 150 mA
Kossen et al. (2023)	p2p GAN TC p2p GAN	MAE NRMSE SSIM PSNR VA	DSC-MRI	1.8 × 1.8 × 5	1.8 × 1.8 × 5	3 T
Olut et al. (2018)	Steerable GAN	PSNR Segmentation	T1-w, T2-w	–	–	1.5 T3 T
Fujita et al. (2020)	U-net TC	SNR PSNR SSIM HFEN VA	3D QALAS MRI	0.5 × 0.5 × 0.5	0.5 × 0.5 × 0.5	3 T
Kossen et al. (2021)	DCGAN WGAN-GP GP-SN	FID Segmentation	Noise vector	–	0.5 × 0.5 × 0.7	3 T
Subramaniam et al. (2022)	WGAN-GP GP-SN GP-SN-MP	FID VA Segmentation	Noise vector	–	0.5 × 0.5 × 0.7	3 T
You et al. (2022)	cycleGAN	SNR PSNR SSIM VA	PETRA-MRA	0.625 × 0.625 × 0.625	0.6 × 0.6 × 0.6	3 T
Kossen et al. (2022)	WGAN-GP GP-SN SN-MP	FID Segmentation	Noise vector	–	0.5 × 0.5 × 0.7	3 T

The table shows characteristics of the networks used to generate medical images. The architecture describes the type of network and the metrics column shows if and how the networks' results were validated and evaluated. FID, The Fréchet Inception distance; MAE, mean absolute error; MAEC, MAE clipped; PVEC, partial volume correction; PSNR, peak signal to noise ration; SSIM, structural similarity index measurement; NRMSE, normalized root mean squared error; corr, the correlation between ground truth and synthetic voxels; *R*^2^, the coefficient of determination; HFEN, the High frequency error norm; VA, visual assessment, are all the metrics used in the studies to assess the performance of the networks. The final column refers to the type of image(s) used to generate either perfusion or MRA images, where DSC-MRI, Dynamic susceptibility contrast-MRI; T1-w, T1-weighted images; T2-w, T2-weighted images; MRF-ASL, MR fingerprinting arterial spin labeling; DA-MRA, contrast-enhanced time-resolved dynamic MR angiography; 3D QALAS MRI, 3D-quantification using an interleaved Look-Locker acquisition sequence with T2 preparation pulse; PETRA-MRA, pointwise encoding time reduction with radial acquisition MRA were used. For the resolution, papers that did not provide the information or where the information was not relevant, such as non-imaging data of the Kossen research group and Zhang et al. (2020), are marked as “–”.

More in line with an assessment of status with respect to the impact in dementia, we found studies that used structural MRI, such as T1-w and T2-w images (e.g., Huang et al., 2019; Li et al., 2021) for image-to-image translation. Similarly, Fujita et al. (2020) used the 3D QALAS sequence, which allows for the coaquisition of T1-, T2-W and FLAIR, to generate TOF-MRA images. These are mostly used due to their ability to provide high-resolution anatomical details of gray and white matter boundaries and detecting structural changes such as brain atrophy.

### 3.6 Methodological quality

Studies from Hess et al. (2019), Zhang et al. (2020), Asaduddin et al. (2023), Gava et al. (2023), Fujita et al. (2020), Kossen et al. (2021), Subramaniam et al. (2022), Kossen et al. (2022) had a dataset containing <200 participants, with Fujita et al. (2020) only having 15 and Zhang et al. (2020) 10 (Table 2). The studies counteracted this by using several data augmentation techniques. For example, Fujita et al. (2020) mentioned 90^*o*^ rotation along the *x* and *y* axis and the addition of Gaussian noise to the images, while Hess et al. (2019) randomly offset the perfusion sequence by −5 to 30 frames by reflecting the sequence. Furthermore, all studies, except Gava et al. (2023) and Zhang et al. (2020) trained their network on 2D or 3D patches of images or sequences to enhance their datasets. Finally, the more focused task of medical image generation and the careful use of data augmentation still led to accurate results, even though the datasets were much smaller than most recent state of the art image generation models, such as DALL-E 2 (650·10^6^ training images) (Ramesh et al., 2022).

Only You et al. (2022), Fujita et al. (2020), Asaduddin et al. (2023), Huang et al. (2019), Li et al. (2021) and Zhang et al. (2020) reported on population age and four of those reported on population sex. The lack of demographics information makes it difficult to evaluate the generalizability of their model in a clinical context.

In addition, many studies used different methods to evaluate the performance of their model. While most studies used metrics commonly used in the field of machine learning, such as FID score, PSNR, SSIM, and MAE, some studies, such as Zhang et al. (2020), used no structural nor error metrics to evaluate their model. Additionally, Asaduddin et al. (2023), Kossen et al. (2023), Kossen et al. (2021), Kossen et al. (2022), Olut et al. (2018), Subramaniam et al. (2022) and the paper by Gava et al. (2023) evaluated their models on other tasks such as vessel segmentation or infarct core segmentation accuracy (either manual or using machine learning). Subramaniam et al. (2022) and Fujita et al. (2020) also used visual assessments by radiologists as a qualitative metric.

## 4 Discussion

### 4.1 Summary

To study the cerebrovascular system in retrospective studies of dementia, new tools must be utilized to counteract the lack of TOF-MRA or perfusion maps in historical datasets or to negate the need to acquire such images in prospective studies. In this review, we identified eleven studies utilizing several different machine learning architectures to synthesize TOF-MRA, perfusion maps, or ASL images. Of those, one study generated ASL images, five generated a variety of perfusion maps (CBF, CBV, Tmax, etc.) and five generated TOF-MRA. All studies, except Hess et al. (2019), managed to consistently generate accurate images. A variety of deep learning models were used in the studies, from simple CNN to U-nets and numerous variations of GANs.

### 4.2 Model architecture and performance

It is difficult to establish which model, if any, is inherently superior, since the tasks to be accomplished and their validation differed greatly. The Kossen research team demonstrated the power of WGAN for the generation of medical images. More precisely, the Subramaniam paper concluded that 3D WGAN with spectral normalization, gradient penalty and mixed precision obtained the best performance. Additionally, their WGAN outclassed the traditional GAN in every instances. On the other hand, You et al. (2022) generated higher quality images. The cycleGAN model demonstrated a greater signal-to-noise ratio than TOF-MRA obtained using a scanner, which is a significant and promising result for the use of machine learning for the study of the cerebrovascular system. Moreover, the SNR, PSNR and SSMI from Fujita and Asaduddin, both using U-net, were all higher than the values from You et al. (2022) which could indicate that the first model is superior, but since both used significantly different training data, such conclusion cannot be verified. Moreover, Asaduddin's study showed U-nets generated more accurate images while GANs generated more structurally sound images while less accurate when compared with the ground truth. This discrepancy is important when trying to generate images for a given subject, but less important when data augmentation is the purpose, like the Kossen team's studies. In terms of the generation of perfusion maps, most authors demonstrated that their models produce comparable results with the state-of-the-art. Gava et al. (2023) generated perfusion maps using only registered CT images and no additional information, as opposed to traditional deconvolution methods that necessitate the arterial input function. Segmentation of the infarct core also showed an accurate representation of the ischemic core, with high Dice score for the validation set (>0.70). On a similar task, Kossen et al.'s temporal pix-2-pix model reported a MAE 34 times lower for generating Tmax maps than the CNN from Hess et al. Additionally, the temporal pix-2-pix outclassed traditional GANs on all metrics (NRMSE, SSIM, and PSNR).

Overall, we could conclude that U-nets would be the best option to generate perfusion or TOF-MRA images when large datasets are available. The main reason for this being that these models produce more accurate images which is an important criterion for image to image translation for diagnosis purposes. Additionally, the use of 3D kernels over 2D improves results as shown by Subramaniam et al. (2022). On the other hand, there is a clear knowledge gap in the use of newer deep learning technologies, such as Transformers and attention networks, although these have been used in the medical field with great success.

### 4.3 Methodological quality

Methodological quality was inconsistent between studies. First, not all studies mentioned data augmentation, which is essential for building a generalized model and when working with smaller datasets. The use of patch learning and other geometric transformations have been known to be effective ways of improving the performance of a model since AlexNet and the dawn of CNN for computer vision (Krizhevsky et al., 2017). It is even more essential for training with low amount of data, which is the case for eight of the eleven articles. Nevertheless, neither Gava et al. (2023) nor Hess et al. (2019) mention geometric augmentation, which could have impacted the generalizability of their technique and thus final validation results.

In addition, most studies used 2D convolutions instead of 3D convolution. For the case of TOF-MRA it is clear that the model from Subramaniam, which uses 3D convolution, obtained better results than the 2D model from the same research team (Kossen et al., 2021, 2022). The results from Fujita et al. (2020) also demonstrated the strength of 3D convolution even for smaller U-net models. For most studies, the reason for not using 3D convolution appears likely to be the lack of computing power, as mentioned in Subramaniam et al. (2022), and Kossen et al. (2022, 2021).

### 4.4 Data considerations

Only Huang et al. (2019), Zhang et al. (2020), Li et al. (2021), Asaduddin et al. (2023), Fujita et al. (2020), You et al. (2022) reported the age and only Zhang et al. (2020), Asaduddin et al. (2023), Fujita et al. (2020), You et al. (2022) reported the sex of the training participants. As previously explained, this discrepancy makes it impossible to evaluate the impact of such variations on the output of the model. Moreover, it was recently exposed that biases in neuroimaging AI models were rampant, both in age and sex, which impacted the performance on trials with more complex data (Chu et al., 2023; Chen et al., 2023). Our research seems to support this general claim of bias in medical AI. Indeed, even though age and sex have been known to be important factors in ischemic stroke, only Asaduddin et al. (2023) of the four papers on that subject provided this information (Roy-O'Reilly and McCullough, 2018; Rexrode et al., 2022). This lack of demographic data limits the evaluation of the models' generalizability, particularly in real-world clinical applications. Models trained on incomplete or homogenous datasets may fail to perform accurately across diverse patient populations, potentially introducing bias in diagnostic outcomes (Singh et al., 2022). In ischemic stroke and other cerebrovascular diseases, for example, sex differences can influence disease progression, recovery, and response to treatment (Rexrode et al., 2022). Furthermore, age-related changes in cerebral hemodynamics are critical when studying neurovascular conditions, as older populations are more prone to vascular pathology (Peters, 2006; Matteis et al., 1998). On a more general note, it is wildly known that the brain undergoes significant physical changes with age, including atrophy; and that the cerebrovascular system weakens with time, which impacts blood flow and increases the probability of vascular diseases (Peters, 2006; Matteis et al., 1998). Because of this, it is obvious that the perfusion maps and arterial structures extracted from TOF-MRA should be impacted by age.

As a result, it becomes crucial for future studies to include comprehensive demographic reporting, and to ensure that machine learning models are evaluated across diverse population subsets. This would allow for a more accurate assessment of the models' utility in predicting perfusion maps and TOF-MRA. Incorporating this data will help validate the robustness of the model when applied to a broader clinical setting, particularly in diseases like Alzheimer's and dementia, where both age and sex significantly influence disease presentation and progression.

Additionally, while the primary focus of this review is on the synthesis of neuroimaging data, it is important to acknowledge that the quality of the original MRI scans can influence the outcomes of synthetic image generation. As with most MRI studies, factors such as image resolution, field strength, and magnetic field homogeneities (or impurities) can significantly impact the quality of the input data. High-resolution scans with consistent field strength generally provide better input for AI models, leading to more accurate synthetic images. However, the studies reviewed did not specifically address the influence of these parameters on image synthesis outcomes. Future research may benefit from exploring how variations in scan quality affect the performance and accuracy of AI models, particularly in generating complex images like TOF-MRA and perfusion MRI.

### 4.5 Input data

The diversity of input data types used across the studies highlights the flexibility of generative models, but also points to the potential advantages of using commonly acquired images like T1-w, T2-w, and FLAIR sequences. These modalities, as seen in studies by Huang et al. (2019), Fujita et al. (2020) and Li et al. (2021), are widely available and easily acquirable. They already provide detailed anatomical information, making them highly valuable for generating synthetic images. The use of these commonly acquired sequences allows for image-to-image translation without the need for specialized or contrast-enhanced scans, potentially making synthetic images more accessible across diverse clinical and research settings. By leveraging these widely used modalities, researchers could generate synthetic images, enabling retrospective studies on previously unexplored datasets in neurovascular diseases and dementia. While the initial results are promising, further research is needed to fully validate the effectiveness of these synthetic images in real-world applications, such as artery segmentation and Alzheimer's disease diagnosis.

### 4.6 Verification, validation, and evaluation

The synthetic perfusion studies we reviewed used different methods to verify, validate, and evaluate their results. After training a machine learning model, it is necessary to verify its coherence, then validate its performance. The best models are tested using validation metrics on a separate dataset than the one used for training. Most of the time verification and validation use the same metrics such as RMSE, FID, SSIM, (P)SNR, and MAE for the field of image synthesis. Evaluation, on the other hand, refers to additional tasks for which the synthesized images bring value, such as vessels or infarct core segmentation, visual assessment, and CBF computation. Table 3 describes the methods used by researchers in the selected studies.

We found discrepancies between projects. Most papers (*n* = 9) used conventional validation metrics, such as MAE, SSIM, SNR, and FID, but two only used evaluation metrics such as infarct core segmentation. The inconsistency between studies renders comparison between results difficult. Only SSIM (*n* = 4), FID (*n* = 3), PSNR (*n* = 3), and MAE (*n* = 2) had cross-over utilization between papers, with FID only being used by the Kossen team. For the evaluation, segmentation of either vessel or infarct core/penumbra (*n* = 5), visual assessment by experts (*n* = 4) and CBF computation (*n* = 1) were the main tasks performed after image generation. Additionally, two papers did not mention splitting the data into training and test sets to get the most accurate results. A good design of image synthesis model should include train/validation/test data, make use of conventional metrics for verification and validation, and be evaluated on a complex task to have the most robust and accurate results.

### 4.7 Limitations

While systematic, this study limited its search to four databases and repositories (PubMed, arXiv, medRxiv, and bioRxiv). This created bias toward English-speaking researchers, which will likely cause this review to be incomplete. Another consideration was the exclusion of super-resolution papers. Indeed, many different studies generated higher resolution TOF-MRA and perfusion from lower-resolution images using machine learning (Wicaksono et al., 2023; Cui et al., 2022). These methods, although integrating images from other modalities such as T1-weighted scans, are usually trained on single images to enhance the resolution of the given image (Shaham et al., 2019). As such, the trained model is not usually generalizable to other images. In that way, it does not fit the definition of image synthesis we used for this review, which specified the use of reusable model.

### 4.8 Conclusion

In this review, we explored the use of deep learning models for the synthesis of TOF-MRA and perfusion MRI images, highlighting the advancements and potential applications in the study of neurovascular health. Our findings suggest that these image synthesis models offer a promising alternative to direct imaging methods, potentially enabling large-scale retrospective analyses and faster coacquisition in new studies and time sensitive interventions, such as stroke disease. Indeed, in most articles reviewed the synthetic images seemed sufficiently accurate to be successfully used to perform higher-value tasks, such as segmenting vessels or infarct cores, and diagnosing dementia. However, the need for comprehensive demographic data, the consideration of model generalizability across diverse populations, and the verification of other sources of bias in the training populations remain critical challenges. Moreover, novel deep learning architectures, such as Transformer, may be better suited at generating synthetic data. Hence, additional studies are necessary to evaluate the viability of TOF-MRA synthesis from commonly acquired data. By addressing these challenges, generated imaging could become a vital tool in advancing our understanding of neurovascular contributions to dementia and other neurological conditions.

## Data Availability

The raw data supporting the conclusions of this article will be made available by the authors, without undue reservation.

## References

[B1] Abrigo J. Chan N. Y. Mok H. Mok V. Kwok T. (2023). Neuroimaging findings in the Hong Kong Alzheimer's disease registry. J. Neurol. Sci. 455:121959. 10.1016/j.jns.2023.12195936795776

[B2] Abu-Srhan A. Almallahi I. Abushariah M. A. M. Mahafza W. Al-Kadi O. S. (2021). Paired-unpaired unsupervised attention guided GAN with transfer learning for bidirectional brain MR-CT synthesis. Comput. Biol. Med. 136:104763. 10.1016/j.compbiomed.2021.104763 34449305

[B3] Albert M. DeKosky S. Salmon D. Morris J. Cairns N. (2010). Alzheimer's Disease Neuroimaging Initiative 2 (ADNI2) Protocol (ADC-039). Available at: https://adni.loni.usc.edu/wp-content/themes/freshnews-dev-v2/documents/clinical/ADNI-2_Protocol.pdf

[B4] Ali H. Biswas M. R. Mohsen F. Shah U. Alamgir A. Mousa O. . (2022). The role of generative adversarial networks in brain MRI: a scoping review. Insights Imaging 13:98. 10.1186/s13244-022-01237-0 35662369 PMC9167371

[B5] Arjovsky M. Chintala S. Bottou L. (2017). Wasserstein GAN. arXiv [preorint]. 10.48550/arXiv.1701.07875

[B6] Asaduddin M. Roh H. G. Kim H. J. Kim E. Y. Park S.-H. (2023). Perfusion maps acquired from dynamic angiography MRI using deep learning approaches. J. Magn. Reson. Imaging 57, 456–469. 10.1002/jmri.28315 35726646

[B7] Bahdanau D. Cho K. Bengio Y. (2016). Neural machine translation by jointly learning to align and translate. arXiv [preprint]. 10.48550/arXiv.1409.0473

[B8] Chen S. Ma K. Zheng Y. (2019). Med3D: transfer learning for 3D medical image analysis. arXiv [preprint]. 10.48550/arXiv.1904.00625

[B9] Chen Z. Liu X. Yang Q. Wang Y.-J. Miao K. Gong Z. . (2023). Evaluation of risk of bias in neuroimaging-based artificial intelligence models for psychiatric diagnosis: a systematic review. JAMA Netw. Open 6:e231671. 10.1001/jamanetworkopen.2023.1671 36877519 PMC9989906

[B10] Chertkow H. Borrie M. Whitehead V. Black S. E. Feldman H. H. Gauthier S. . (2019). The comprehensive assessment of neurodegeneration and dementia: Canadian Cohort Study. Can. J. Neurol. Sci. 46, 499–511. 10.1017/cjn.2019.27 31309917

[B11] Chu C. H. Donato-Woodger S. Khan S. S. Nyrup R. Leslie K. Lyn A. . (2023). Age-related bias and artificial intelligence: a scoping review. Human. Soc. Sci. Commun. 10, 1–17. 10.1057/s41599-023-01999-y

[B12] Cui J. Gong K. Han P. Liu H. Li Q. (2022). Unsupervised arterial spin labeling image superresolution via multiscale generative adversarial network. Med. Phys. 49, 2373–2385. 10.1002/mp.15468 35048390

[B13] de la Torre J. C. (1999). Critical threshold cerebral hypoperfusion causes Alzheimer's disease? Acta Neuropathol. 98, 1–8. 10.1007/s004010051044 10412794

[B14] Duchesne S. Rousseau L.-S. Belzile F. Welsh L.-A. Cournoyer B. Arseneau M. . (2023). A scoping review of Alzheimer's disease hypotheses: the case for a multi-factorial theory. medrxiv. [preprint]. 10.1101/2023.07.26.23293030

[B15] Essig M. Shiroishi M. S. Nguyen T. B. Saake M. Provenzale J. M. Enterline D. . (2013). Perfusion MRI: the five most frequently asked technical questions. Am. J. Roentgenol. 200, 24–34. 10.2214/AJR.12.9543 23255738 PMC3593114

[B16] Fujita S. Hagiwara A. Otsuka Y. Hori M. Takei N. Hwang K.-P. . (2020). Deep learning approach for generating MRA images from 3D quantitative synthetic MRI without additional scans. Invest. Radiol. 55, 249–256. 10.1097/RLI.0000000000000628 31977603

[B17] Gaillard F. (2016). Dynamic susceptibility contrast (DSC) MR perfusion. Radiopaedia. 10.53347/rID-43780

[B18] Gava U. A. D'Agata F. Tartaglione E. Renzulli R. Grangetto M. Bertolino F. . (2023). Neural network-derived perfusion maps: a model-free approach to computed tomography perfusion in patients with acute ischemic stroke. Front. Neuroinform. 17:852105. 10.3389/fninf.2023.852105 36970658 PMC10034033

[B19] Goodfellow I. Pouget-Abadie J. Mirza M. Xu B. Warde-Farley D. Ozair S. . (2014). Generative adversarial networks. arXiv [preprint]. 10.48550/arXiv.1406.2661

[B20] Gunter J. L. Borowski B. J. Thostenson K. Arani A. Reid R. I. Cash D. M. . (2017). [ic-P-137]: Adni-3 MRI protocol. Alzheimers Dement. 13, P104–P105. 10.1016/j.jalz.2017.06.2411

[B21] He K. Gan C. Li Z. Rekik I. Yin Z. Ji W. . (2022). Transformers in medical image analysis: a review. arXiv [preprint]. 10.48550/arXiv.2202.12165

[B22] Hess A. Meier R. Kaesmacher J. Jung S. Scalzo F. Liebeskind D. . (2019). Synthetic perfusion maps: Imaging perfusion deficits in DSC-MRI with deep learning. IEEE Trans. Med. Imaging 38, 2338–2351. 10.1109/TML.2019.290667730908201

[B23] Hotter B. Pittl S. Ebinger M. Oepen G. Jegzentis K. Kudo K. . (2009). Prospective study on the mismatch concept in acute stroke patients within the first 24 h after symptom onset - 1000Plus study. BMC Neurol. 9:60. 10.1186/1471-2377-9-60 19995432 PMC3224745

[B24] Huang W. Luo M. Liu X. Zhang P. Ding H. Xue W. . (2019). Arterial spin labeling images synthesis from sMRI using unbalanced deep discriminant learning. IEEE Transact. Med. Imaging 38, 2338–2351. 10.1109/TMI.2019.2906677 30908201

[B25] Innovation V. H. (2023). Covidence - *Better Systematic Review Management*. Covidence.

[B26] Iturria-Medina Y. Sotero R. C. Toussaint P. J. Mateos-P?rez J. M. Evans A. C. (2016). Early role of vascular dysregulation on late-onset Alzheimer's disease based on multifactorial data-driven analysis. Nat. Commun. 7:11934. 10.1038/ncomms11934 27327500 PMC4919512

[B27] Jack C. R. Jr. Bernstein M. A. Fox N. C. Thompson P. Alexander G. Harvey D. . (2008). The Alzheimer's Disease Neuroimaging Initiative (ADNI): MRI methods. J. Magn. Reson. Imaging 27, 685–691. 10.1002/jmri.21049 18302232 PMC2544629

[B28] Kalaria R. N. (1992). The blood-brain barrier and cerebral microcirculation in Alzheimer disease. Cerebrovasc. Brain Metab. Rev. 4, 226–260. 1389957

[B29] Kazeminia S. Baur C. Kuijper A. van Ginneken B. Navab N. Albarqouni S. . (2020). GANs for medical image analysis. Artif. Intell. Med. 109:101938. 10.1016/j.artmed.2020.101938 34756215

[B30] Koenig M. Klotz E. Luka B. Venderink D. J. Spittler J. F. Heuser L. (1998). Perfusion CT of the brain: diagnostic approach for early detection of ischemic stroke. Radiology 209, 85–93. 10.1148/radiology.209.1.9769817 9769817

[B31] Kossen T. Hirzel M. A. Madai V. I. Boenisch F. Hennemuth A. Hildebrand K. . (2022). toward sharing brain images: differentially private TOF-mra images with segmentation labels using generative adversarial networks. Front. Artif. Intell. 5:813842. 10.3389/frai.2022.813842 35586223 PMC9108458

[B32] Kossen T. Madai V. I. Mutke M. A. Hennemuth A. Hildebrand K. Behland J. . (2023). Image-to-image generative adversarial networks for synthesizing perfusion parameter maps from DSC-MR images in cerebrovascular disease. Front. Neurol. 13:1051397. 10.3389/fneur.2022.1051397 36703627 PMC9871486

[B33] Kossen T. Subramaniam P. Madai V. I. Hennemuth A. Hildebrand K. Hilbert A. . (2021). Synthesizing anonymized and labeled TOF-MRA patches for brain vessel segmentation using generative adversarial networks. Comput. Biol. Med. 131:104254. 10.1016/j.compbiomed.2021.104254 33618105

[B34] Krizhevsky A. Sutskever I. Hinton G. E. (2017). Imagenet classification with deep convolutional neural networks. Commun. ACM 60, 84–90. 10.1145/3065386

[B35] LaMontagne P. J. Benzinger T. L. Morris J. C. Keefe S. Hornbeck R. Xiong C. . (2019). OASIS-3: longitudinal neuroimaging, clinical, and cognitive dataset for normal aging and *Alzheimer Disease*. 10.1101/2019.12.13.19014902

[B36] Laub G. A. (1995). Time-of-flight method of MR angiography. Magn. Reson. Imaging Clin. N. Am. 3, 391–398. 10.1016/S1064-9689(21)00251-8 7584245

[B37] Li F. Huang W. Luo M. Zhang P. Zha Y. (2021). A new VAE-GAN model to synthesize arterial spin labeling images from structural MRI. Displays 70:102079. 10.1016/j.displa.2021.102079

[B38] Luo X. Li K. Zeng Q. Huang P. Jiaerken Y. Wang S. . (2019). Application of T1-/T2-weighted ratio mapping to elucidate intracortical demyelination process in the Alzheimer's disease continuum. Front. Neurosci. 13:904. 10.3389/fnins.2019.00904 31551678 PMC6748350

[B39] Mann D. M. (1985). The neuropathology of Alzheimer's disease: a review with pathogenetic, aetiological and therapeutic considerations. Mech. Ageing Dev. 31, 213–255. 10.1016/0047-6374(85)90092-2 3906293

[B40] Manzari O. N. Ahmadabadi H. Kashiani H. Shokouhi S. B. Ayatollahi A. (2023). MedViT: A robust vision transformer for generalized medical image classification. Comput. Biol. Med. 157:106791. 10.1016/j.compbiomed.2023.106791 36958234

[B41] Matteis M. Troisi E. Monaldo B. C. Caltagirone C. Silvestrini M. (1998). Age and sex differences in cerebral hemodynamics. Stroke 29, 963–967. 10.1161/01.STR.29.5.963 9596243

[B42] Mehrabi N. Morstatter F. Saxena N. Lerman K. Galstyan A. (2021). A survey on bias and fairness in machine learning. ACM Comp. Surv. 54:115. 10.1145/3457607

[B43] Miyazaki M. Akahane M. (2012). Non-contrast enhanced MR angiography: established techniques. J. Magn. Reson. Imaging 35, 1–19. 10.1002/jmri.22789 22173999

[B44] Mutke M. A. Madai V. I. Samson-Himmelstjerna F. C. Weber O. Z. Revankar G. S. Martin S. Z. . (2014). Clinical evaluation of an arterial-spin-labeling product sequence in steno-occlusive disease of the brain. PLoS ONE, 9:e87143. 10.1371/journal.pone.0087143 24516546 PMC3916330

[B45] Olut S. Sahin Y. H. Demir U. Unal G. (2018). “Generative adversarial training for MRA image synthesis using multi-contrast MRI,” in PRedictive Intelligence in MEdicine, eds. I. Rekik, G. Unal, E. Adeli, and S. H. Park (Cham: Springer International Publishing), 147–154.

[B46] Page M. J. McKenzie J. E. Bossuyt P. M. Boutron I. Hoffmann T. C. Mulrow C. D. . (2021). The PRISMA 2020 statement: an updated guideline for reporting systematic reviews. BMJ 372:n71. 10.31222/osf.io/v7gm2 33782057 PMC8005924

[B47] Parkes L. M. Rashid W. Chard D. T. Tofts P. S. (2004). Normal cerebral perfusion measurements using arterial spin labeling: reproducibility, stability, and age and gender effects. Magn. Reson. Med. 51, 736–743. 10.1002/mrm.20023 15065246

[B48] Peters R. (2006). Ageing and the brain. Postgrad. Med. J. 82, 84–88. 10.1136/pgmj.2005.036665 16461469 PMC2596698

[B49] Petrella J. R. Provenzale J. M. (2000). MR perfusion imaging of the brain: techniques and applications. Am. J. Roentgenol. 175, 207–219. 10.2214/ajr.175.1.1750207 10882275

[B50] Prosser L. (2024). Investigating Imaging Biomarkers in Normal Ageing and Cognitive Impairment (Doctoral thesis). London: UCL (University College London).

[B51] Ramesh A. Dhariwal P. Nichol A. Chu C. Chen M. (2022). Hierarchical text-conditional image generation with CLIP latents. arXiv [preprint]. 10.48550/arXiv.2204.06125

[B52] Rexrode K. M. Madsen T. E. Yu A. Y. Carcel C. Lichtman J. H. Miller E. C. (2022). The impact of sex and gender on stroke. Circ. Res. 130, 512–528. 10.1161/CIRCRESAHA.121.319915 35175851 PMC8890686

[B53] Ronneberger O. Fischer P. Brox T. (2015). U-Net: convolutional networks for biomedical image segmentation. arXiv [preprint[]. 10.1007/978-3-319-24574-4_28

[B54] Roy-O'Reilly M. McCullough L. D. (2018). Age and sex are critical factors in ischemic stroke pathology. Endocrinology 159, 3120–3131. 10.1210/en.2018-00465 30010821 PMC6963709

[B55] Shaham T. R. Dekel T. Michaeli T. (2019). SinGAN: learning a generative model from a single natural image. arXiv [preprint]. 10.1109/ICCV.2019.00467

[B56] Shamshad F. Khan S. Zamir S. W. Khan M. H. Hayat M. Khan F. S. . (2023). Transformers in medical imaging: a survey. Med. Image Anal. 88:102802. 10.1016/j.media.2023.102802 37315483

[B57] Singh H. Mhasawade V. Chunara R. (2022). Generalizability challenges of mortality risk prediction models: a retrospective analysis on a multi-center database. PLOS Digit. Health 1:e0000023. 10.1371/journal.pdig.0000023 36812510 PMC9931319

[B58] Smith M. Alfaro-Almagro S. Miller K. (2014). UK Biobank Brain Imaging Documentation. Available at: https://biobank.ctsu.ox.ac.uk/crystal/crystal/docs/brain_mri.pdf

[B59] Subramaniam P. Kossen T. Ritter K. Hennemuth A. Hildebrand K. Hilbert A. . (2022). Generating 3D TOF-MRA volumes and segmentation labels using generative adversarial networks. Med. Image Anal. 78:102396. 10.1016/j.media.2022.102396 35231850

[B60] Szegedy C. Vanhoucke V. Ioffe S. Shlens J. Wojna Z. (2015). Rethinking the inception architecture for computer vision. arXiv [preprint]. 10.1109/CVPR.2016.308

[B61] Ueda T. Hatakeyama T. Kumon Y. Sakaki S. Uraoka T. (1994). Evaluation of risk of hemorrhagic transformation in local intra-arterial thrombolysis in acute ischemic stroke by initial SPECT. Stroke 25, 298–303. 10.1161/01.STR.25.2.298 8303735

[B62] Ueda T. Yuh W. T. Maley J. E. Otake S. Quets J. P. Taoka T. . (1999). Current and future imaging of acute cerebral ischemia: assessment of tissue viability by perfusion imaging. J. Comput. Assist. Tomogr. 23(Suppl. 1):S3–7. 10.1097/00004728-199911001-00002 10608392

[B63] Vaswani A. Shazeer N. Parmar N. Uszkoreit J. Jones L. Gomez A. N. . (2017). Attention is all you need. arXiv [preprint]. 10.48550/arXiv.1706.03762

[B64] Walker M. Deshmukh S. Harbison D. Partovi S. (2001). CT perfusion imaging. Barrow Neurol. Inst. 17.

[B65] Wheaton A. J. Miyazaki M. (2012). Non-contrast enhanced MR angiography: physical principles. J. Magn. Reson. Imaging 36, 286–304. 10.1002/jmri.23641 22807222

[B66] Wicaksono K. P. Fujimoto K. Fushimi Y. Sakata A. Okuchi S. Hinoda T. . (2023). Super-resolution application of generative adversarial network on brain time-of-flight MR angiography: image quality and diagnostic utility evaluation. Eur. Radiol. 33, 936–946. 10.1007/s00330-022-09103-9 36006430

[B67] Yi X. Walia E. Babyn P. (2019). Generative adversarial network in medical imaging: a review. Med. Image Anal. 58:101552. 10.1016/j.media.2019.101552 31521965

[B68] You S.-H. Cho Y. Kim B. Yang K.-S. Kim B. K. Park S. E. (2022). Synthetic time of flight magnetic resonance angiography generation model based on cycle-consistent generative adversarial network using PETRA-MRA in the patients with treated intracranial aneurysm. J. Magn. Reson. Imaging 56, 1513–1528. 10.1002/jmri.28114 35142407

[B69] Zhang Q. Su P. Chen Z. Liao Y. Chen S. Guo R. . (2020). Deep learning-based MR fingerprinting ASL ReconStruction (DeepMARS). Magn. Reson. Med. 84, 1024–1034. 10.1002/mrm.28166 32017236

[B70] Zhao J. Hou X. Pan M. Zhang H. (2022). Attention-based generative adversarial network in medical imaging: a narrative review. Comput. Biol. Med. 149:105948. 10.1016/j.compbiomed.2022.105948 35994931

[B71] Zhu J.-Y. Park T. Isola P. Efros A. A. (2020). Unpaired image-to-image translation using cycle-consistent adversarial networks. arXiv [preprint]. 10.48550/arXiv.1703.10593

